# Effects of Osmolality on Paracellular Transport in MDCK II Cells

**DOI:** 10.1371/journal.pone.0166904

**Published:** 2016-11-17

**Authors:** Shinsaku Tokuda, Toyohiro Hirai, Mikio Furuse

**Affiliations:** 1 Department of Respiratory Medicine, Graduate School of Medicine, Kyoto University, Kyoto, Japan; 2 Division of Cell Structure, National Institute for Physiological Sciences, Okazaki, Japan; 3 Department of Physiological Sciences, SOKENDAI (The Graduate University for Advanced Studies), Okazaki 444–8585, Japan; Emory University School of Medicine, UNITED STATES

## Abstract

Epithelia separate apical and basal compartments, and movement of substances via the paracellular pathway is regulated by tight junctions. Claudins are major constituents of tight junctions and involved in the regulation of tight junction permeability. On the other hand, the osmolality in the extracellular environment fluctuates in association with life activity. However, effects of osmotic changes on the permeaibility of claudins are poorly understood. Therefore, we investigated the effects of osmotic changes on the paracellular transport in MDCK II cells. Interestingly, apical hyposmolality decreased cation selectivity in the paracellular pathway gradually with time, and the elimination of the osmotic gradient promptly restored the cation selectivity. Apical hyposmolality also induced bleb formation at cell-cell contacts and changed the shape of cell-cell contacts from a jagged pattern to a slightly linear pattern. In claudin-2 knockout MDCK II cells, the decrease of cation selectivity, the bleb formation, nor the changes in the shape of cell-cell contacts was observed under the apical hyposmolality. Our findings in this study indicate that osmotic gradient between apical and basal sides is involved in the acute regulation of the cation selective property of claudin-2 channels.

## Introduction

In multicellular organisms, epithelia act as a barrier between the external and internal environment. There are two routes for the movement of substances across the epithelia: transcellular and paracellular pathways. The permeability of the paracellular pathway is regulated by tight junctions (TJs), which are one mode of the junctional complexes located in the most apical part of the complexes [1–4]. On the other hand, the osmolality in the extracellular environment fluctuates in association with life activity such as water intake. However, there have not been many reports that studied the effects of osmolality on the paracllular transport [5], and the regulatory mechanism of the paracellular transport by the osmolality was incompletely clarified.

Claudins, a large family of integral membrane proteins constituting TJ strands, are the major determinants of TJ permeability [6–8]. Epithelial cells express multiple different claudins, and the expression pattern of claudins provides a variety of TJ permeabilities [9,10]. After the identification of claudins in 1998, osmotic changes have been reported to affect claudin expression pattern in euryhaline fishs and cultured cells [11–15]. However, effects of osmotic changes on the permeaibility of claudin channels are poorly understood.

The transport properties of claudin-2 have been particularly well studied. Claudin-2 forms highly conductive channels with cation selectivity in TJs [16–18]. Madin-Darby canine kidney (MDCK) II cells express claudin-2 [19] and the property of paracellular transport is well studied. Therefore, in this study, we used MDCK II cells and investigated the effects of osmotic changes in the apical and basal sides on the paracellular transport. Our findings indicate that osmotic gradient between apical and basal sides is involved in the acute regulation of paracellular transport.

## Results

### Effects of hyposmolality on the barrier function in MDCK II cells

**Fig 1:**
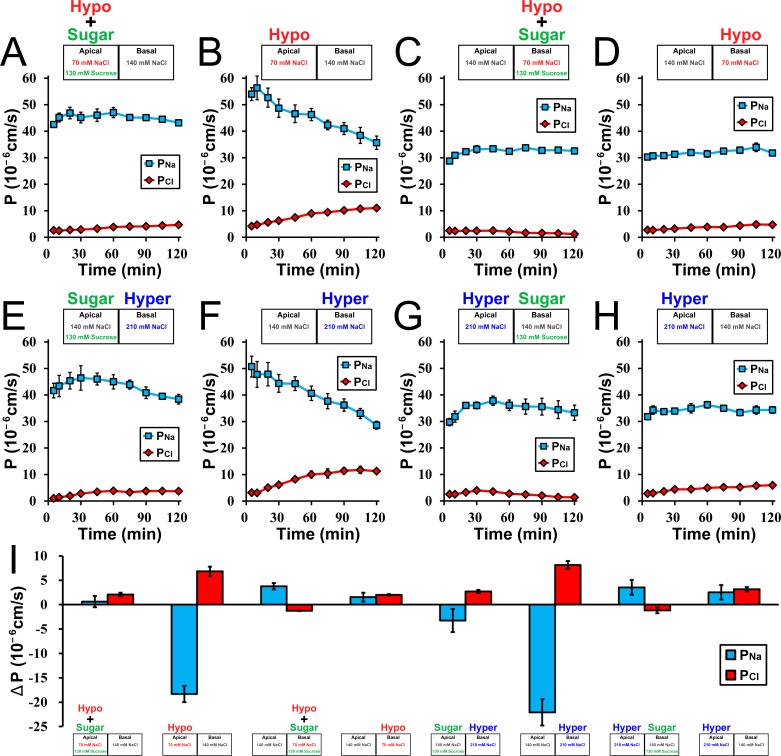
Effects of osmolality on the barrier function in MDCK II cells. (A–H) Time course of *P*_*Na*_ and *P*_*Cl*_ in MDCK II cells. Apical and/or basal solutions were replaced at time 0, and the composition of NaCl and sucrose in the replaced solutions is shown in the upper sides of each graph. *P*_*Na*_ and *P*_*Cl*_ were measured at each time point during 120 min. (I) The variations of *P*_*Na*_ and *P*_*Cl*_ during 115 min under the osmotic changes. The values of *P*_*Na*_ and *P*_*Cl*_ at time 120 min were subtracted from those at time 5 min, respectively. The composition of NaCl and sucrose in the solutions in each condition is shown in the lower sides of the bars. The values of *P*_*Na*_ were decreased and *P*_*Cl*_ were increased during 115 min under the apical hyposmolality and basal hyperosmolality. N = 3–4 for each experiment.

To study the effects of osmotic changes on the paracellular transport in MDCK II cells, we measured the transepithelial ion permeability of Na^+^ and Cl^-^ across the epithelia (*P*_*Na*_ and *P*_*Cl*_) by changing NaCl concentration and osmolality in the buffer solutions. First, we investigated the effects of hyposmolality on *P*_*Na*_ and *P*_*Cl*_ in MDCK II cells. Under the condition where NaCl concentration in the apical side was decreased by half and the osmolality was adjusted with sucrose (‘apical isosmotic’ condition), the value of *P*_*Na*_ was much higher than *P*_*Cl*_ immediately after the replacement of the apical solution, and the values of *P*_*Na*_ and *P*_*Cl*_ were almost constant during 120 min of incubation (Fig 1A). In contrast, under the condition where NaCl concentration in the apical side was decreased by half and the osmolality was not adjusted (‘apical hyposmotic’ condition), the value of *P*_*Na*_ was also much higher than *P*_*Cl*_ immediately after the replacement of the apical solution, but then the *P*_*Na*_ decreased and *P*_*Cl*_ increased gradually with time (Fig 1B). The cation selectivity (ratio of *P*_*Na*_ to *P*_*Cl*_: *P*_*Na*_/*P*_*Cl*_) in the ‘apical hyposmotic’ condition at 120 min after the incubation was significantly lower than that in the ‘apical isosmotic’ condition (‘apical isosmotic’ condition, 9.32 ± 0.51 vs ‘apical hyposmotic’ condition, 3.24 ± 0.08). The decrease of cation selectivity was also not observed under the condition where apical osmolality was adjusted with mannitol in place of sucrose (S1A Fig), indicating the suppression of the decrease of cation selectivity under the ‘apical isosmotic’ condition is not due to the specific action of sucrose. On the other hand, under the ‘basal isosmotic’ and ‘basal hyposmotic’ conditions, the values of *P*_*Na*_ were also much higher than *P*_*Cl*_, and these values were almost constant during 120 min (Fig 1C and 1D). These results indicate that apical hyposmolality induces a gradual decrease in *P*_*Na*_ and an increase in *P*_*Cl*_ in MDCK II cells.

### Effects of hyperosmolality on the barrier function in MDCK II cells

The decrease in osmolality in the apical side under the ‘apical hyposmotic’ condition is thought to generate osmotic gradient between apical and basal sides, which is likely to decrease the cation selectivity in MDCK II cells. To study this possibility, we investigated the effects of hyperosmolality on *P*_*Na*_ and *P*_*Cl*_ in MDCK II cells. Under the condition where NaCl concentration in the basal side was increased (‘basal hyperosmotic’ condition), the value of *P*_*Na*_ decreased and *P*_*Cl*_ increased gradually with time similar to those in the ‘apical hyposmotic’ condition (Fig 1F). The addition of sucrose to the apical side to counterbalance the osmotic gradient between apical and basal sides suppressed these changes (Fig 1E). Under the condition where NaCl concentration in the apical side was increased, the values of *P*_*Na*_ and *P*_*Cl*_ were almost constant during 120 min regardless of the addition of sucrose to the basal side (Fig 1G and 1H). These results indicate that basal hyperosmolality also induces a gradual decrease in *P*_*Na*_ and increase in *P*_*Cl*_ in MDCK II cells, and the relative apical hyposmolality (osmotic gradient between apical and basal sides) causes the decrease of cation selectivity in MDCK II cells (Fig 1I).

### Effects of osmolality on the localization of TJ proteins in MDCK II cells

**Fig 2:**
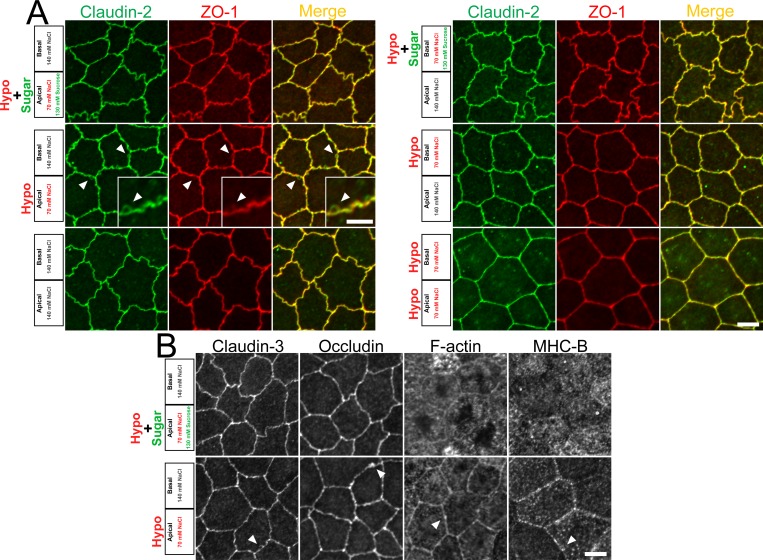
Effects of hyposmolality on the localization of TJ proteins and cytoskeleton. (A) Immunofluorescence microscopy for claudin-2 and ZO-1 in a horizontal section at apical junctional levels. Epithelia were fixed 30 min after the osmotic changes. The composition of NaCl and sucrose in the solutions in each condition is shown on the left sides of the images. Under the apical hyposmolality, cell-cell contacts showed a slightly linear shape, and protruded signals of claudin-2 and ZO-1 from the lines of cell-cell contacts were observed (*arrowheads*). (B) Immunofluorescence microscopy for claudin-3, occludin, F-actin, and myosin heavy chain II-B (MHC-B). The protruded signals of claudin-3, occludin, F-actin and MHC-B were observed under the apical hyposmolality (*arrowheads*). Scale bars = 5 μm.

In the so-called ‘leaky’ epithelia that have low transepithelial resistance such as MDCK II cells, the ion permeability across the epithelia is mostly determined by the permeability of TJs in the paracellular pathway [20,21]. Since claudin-2 has been reported to be a major determinant of the high transepithelial conductance and cation selectivity in MDCK II cells [19,22], we investigated the effects of osmolality on the localization of claudin-2. The epithelia were fixed 30 min after the replacement of the solutions, and signals of claudin-2 and ZO-1 (scaffold protein in TJs [23]) were observed by immunofluorescence microscopy. Under the condition where MDCK II cells were incubated in normal buffer solutions for 30 min (control experiment), the signals of claudin-2 and ZO-1 at apical junctional levels showed a jagged shape of cell-cell contacts in MDCK II cells (Fig 2A, lower-left panels). The localization of claudin-2 and ZO-1 under the ‘apical isosmotic’ condition was similar to that under the control condition (Fig 2A, upper-left panels and S1B Fig). In contrast, under the ‘apical hyposmotic’ condition, the cell-cell contacts showed a slightly linear shape compared with the control condition, and protruded signals of claudin-2 and ZO-1 from the lines of cell-cell contacts were observed (Fig 2A, middle-left panels and S2 Fig). The protrusions were more noticeable in the signals of claudin-2 than ZO-1. On the other hand, the protruded signals of claudin-2 and ZO-1 were not observed under the ‘basal hyposmotic’ condition and both of apical and basal hyposmotic condition, although the cell-cell contacts showed a slightly linear shape under these conditions (Fig 2A, middle- and lower-right panels).

To investigate the effects of apical hyposmolality on the localization of other TJ proteins and cytoskeleton, we examined the localization of claudin-3, occludin (integral membrane protein in TJs), F-actin, and myosin heavy chain II-B (MHC-B). The protruded signals from the lines of cell-cell contacts were also observed in claudin-3, occludin, F-actin and MHC-B signals under the ‘apical hyposmotic’ condition (Fig 2B). These results suggest that apical hyposmolality alters the localization of TJ proteins and cytoskeleton at cell-cell contacts in MDCK II cells.

**Fig 3:**
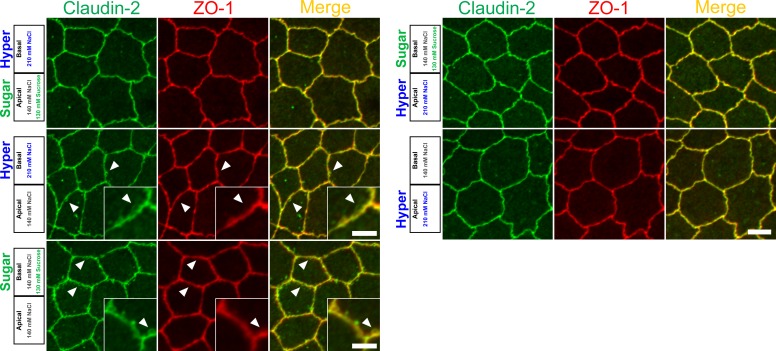
Effects of hyperosmolality on the localization of claudin-2 and ZO-1. Immunofluorescence microscopy for claudin-2 and ZO-1. Under the basal hyperosmolality with NaCl or sucrose, protruded signals of claudin-2 and ZO-1 from the lines of cell-cell contacts were observed (*arrowheads*). Scale bars = 5 μm.

We further investigated the effects of hyperosmolality on the localization of claudin-2 and ZO-1 in MDCK II cells. The protruded signals of claudin-2 and ZO-1 were also observed under the ‘basal hyperosmotic’ condition (Fig 3, middle-left panels), and the addition of sucrose to the apical side to counterbalance the osmotic gradient suppressed these changes (Fig 3, upper-left panels). Interestingly, the addition of sucrose to the basal side to increase the osmolality in the basal side also induced the protrusion of claudin-2 and ZO-1 signals (Fig 3, lower-left panels). These changes were not observed under the apical hyperosmotic condition (Fig 3, right panels). These results indicate that relative apical hyposmolality alters the localization of claudin-2 and ZO-1 at cell-cell contacts in MDCK II cells.

### Effects of osmolality on the surface structure of MDCK II cells

**Fig 4:**
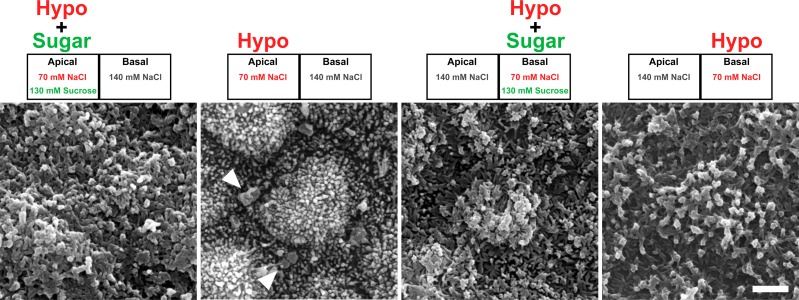
Scanning electron microscopy of MDCK II cells under the hyposmolality. Epithelia were fixed 30 min after the osmotic changes and observed by scanning electron microscopy. The composition of NaCl and sucrose in the solutions in each condition is shown in the upper sides of the images. Globular structures were observed around cell-cell contacts under the apical hyposmolality (*arrowheads*). Scale bar = 2 μm.

Next, we further examined the structural changes at cell-cell contacts in the relative apical hyposmolality. MDCK II cells were fixed at 30 min after the application of osmotic changes and observed by scanning electron microscopy. Under the ‘apical isosmotic’ condition, the cell surface of MDCK II cells showed slightly raised apical cell membranes with microvilli and groove-like cell-cell contact regions (Fig 4 and S3 Fig). In contrast, we found globular structures at cell-cell contact regions under the ‘apical hyposmotic’ condition. The globular structures were around 1 μm in diameter and showed inflated or deflated appearance (Fig 4 and S3 Fig). Similar globular structures were also observed under the basal hyperosmolality, although the number of the structures was smaller than that under the apical hyposmolality (S3 and S4 Figs). These results indicate that relative apical hyposmolality induces the formation of globular structures at cell-cell contact regions in MDCK II cells.

### Apical hyposmolality induces dynamic changes in claudin-2 and F-actin localization in MDCK II cells

**Fig 5:**
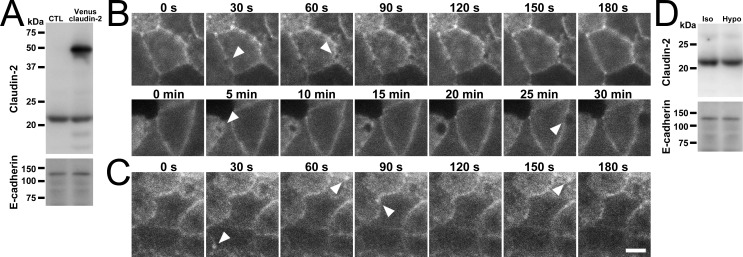
Time-lapse imaging of MDCK II cells under the apical hyposmolality. (A) Immunoblots for claudin-2 and E-cadherin in control and Venus-claudin-2 expressing MDCK II cells. The MDCK II cell clone expressing Venus-claudin-2 was established, and the band of Venus-claudin-2 was detected at the expected molecular weight in addition to endogenous claudin-2 bands. (B) Time-lapse imaging of claudin-2 under the apical hyposmolality. The images of fluorescent Venus signal were collected immediately after the application of osmotic changes every 30 sec. The signal of claudin-2 showed the occurrence of low signal circular structures at various regions in cell-cell contacts, and these structures expanded to a diameter of about one to three μm and then disappeared during 30 sec to several minutes (*arrowheads*). (C) Time-lapse imaging of Lifeact under the apical hyposmolality. The signal of Lifeact showed similar dynamic changes in claudin-2 (*arrowheads*). (D) Effects of apical hyposmolality on the amount of claudin-2 proteins. MDCK cells were collected 120 min after the application of osmotic changes, and immunoblot analysis for claudin-2 and E-cadherin was performed. A protein expression level of claudin-2 under the ‘apical hyposmotic’ condition (Hypo) was similar to that under the ‘apical isosmotic’ condition (Iso). Scale bar = 5 μm.

To investigate the changes in the localization of TJ proteins and globular structures observed under the apical hyposmolality in detail, we established a stable clone of MDCK II cells expressing canine claudin-2 cDNA tagged with a fluorescent protein Venus (Fig 5A) and performed time-lapse imaging. Under the ‘apical isosmotic’ condition, the Venus signal of claudin-2 showed modest sequential changes during 30 min of the observation (S1 Movie). In contrast, the signal of claudin-2 under the ‘apical hyposmotic’ condition showed the occurrence of low signal circular structures at various regions in cell-cell contacts (Fig 5B and S2 and S3 Movies). These structures expanded to a diameter of about one to three μm and then disappeared within 30 sec to several minutes. We also established a stable clone expressing Venus Lifeact (F-actin marker for higher eukaryotes [24]) and performed time-lapse imaging. The signal of Lifeact under the ‘apical hyposmotic’ condition also showed similar dynamic changes observed in claudin-2, although the signal intensity of Lifeact in the circular structures was high (Fig 5C and S4–S6 Movies). These results indicate that the apical hyposmolality triggers dynamic occurrence and disappearance of bleb-like structures at cell-cell contacts in MDCK II cells. In addition, the changes observed in immunofluorescence microscopy and scanning electron microscopy are likely to reflect the dynamic changes in claudin-2 and F-actin localization observed in time-lapse imaging.

To confirm the effects of apical hyposmolality on the shape of cell-cell contacts, we also established a stable clone expressing Venus occludin and compared the signal of occludin before and 120 min after the apical hyposmolality. We confirmed that the jagged pattern of cell-cell contacts was changed to a slightly linear pattern by the apical hyposmolality (S5 Fig).

We also examined whether the amount of claudin-2 protein was changed in the apical hyposmolality. In immunoblot analysis of MDCK II cells collected 120 min after the application of osmotic changes, the protein expression level of claudin-2 under the ‘apical hyposmotic’ condition was similar to that under the ‘apical isosmotic’ condition (Fig 5D). These results suggest that the ‘apical hyposmotic’ condition does not cause an obvious change in the amount of claudin-2 protein in MDCK II cells.

### Bleb-like structure induced by the apical hyposmolality occurs at immediate vicinity of TJ strands

**Fig 6:**
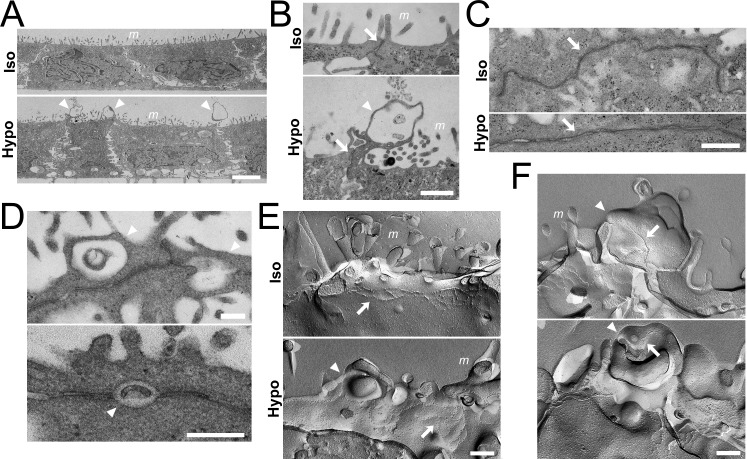
Transmission and freeze-fracture electron microscopy of MDCK II cells under the apical hyposmolality. (A–C) Transmission electron microscopy of MDCK II cells under the ‘apical isosmotic’ (Iso) and ‘apical hyposmotic’ (Hypo) conditions in a vertical section (A and B) and a horizontal section (C). Epithelia were fixed 30 min after the osmotic changes. Bleb-like structures were observed in the immediate vicinity of TJs under the ‘apical hyposmotic’ condition (*arrowheads*), and miscellaneous structures were observed in the blebs. Cell-cell contacts at TJ level showed a linear shape under the ‘apical hyposmotic’ condition compared with the ‘apical isosmotic’ condition (*arrows*). Scale bars = 2 μm for (A), 500 nm for (B), and 200 nm for (C). (D) Transmission electron microscopy of MDCK II cells under the ‘apical hyposmotic’ condition in a horizontal section. Blebs were observed in the immediate vicinity of TJs (*arrowheads*). Scale bars = 200 nm. (E) Freeze-fracture electron microscopy of MDCK II cells under the ‘apical isosmotic’ condition (Iso) and ‘apical hyposmotic’ condition (Hypo). The blebs were observed in the vicinity of TJ strands under the ‘apical hyposmotic’ condition (*arrowheads*). Scale bar = 200nm. (F) Freeze-fracture electron microscopy of MDCK II cells under the ‘apical hyposmotic’ condition. TJ strands were occasionally found on the blebs (*arrows*). Scale bar = 200nm. *m*, microvilli.

To observe the bleb-like structures and TJs in the apical hyposmolality in detail, we performed transmission electron microscopy in MDCK II cells fixed at 30 min after the application of osmotic changes. In a vertical section, bleb-like structures were observed in the vicinity of TJs under the ‘apical hyposmotic’ condition, and miscellaneous structures were observed in the blebs (Fig 6A and 6B). In a horizontal section, cell-cell contacts at TJ level showed a linear shape under the ‘apical hyposmotic’ condition compared with the ‘apical isosmotic’ condition, and the blebs were observed in the immediate vicinity of TJs (Fig 6C and 6D). We further performed freeze-fracture electron microscopy to observe the blebs and TJ strands. Under the ‘apical isosmotic’ condition, anastomosing particle strands (TJ strands) were observed below the microvilli (Fig 6E). In contrast, we found the blebs in the vicinity of TJ strands under the ‘apical hyposmotic’ condition (Fig 6E), and the strands were occasionally observed on the blebs (Fig 6F). These results indicate that the blebs induced by the apical hyposmolality arise at the immediate vicinity of TJ strands in MDCK II cells.

### Reversibility of the changes induced by the apical hyposmolality

**Fig 7:**
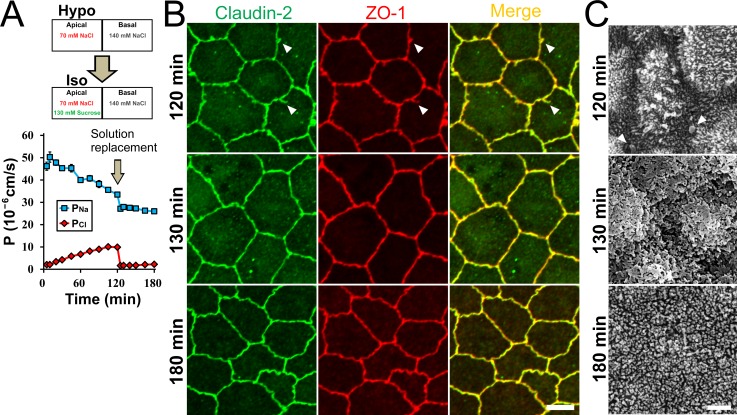
Reversibility of the changes induced by the apical hyposmolality. (A) Time course of *P*_*Na*_ and *P*_*Cl*_. MDCK II cells were incubated in the ‘apical hyposmotic’ condition for 120 min, and then the condition was changed to the ‘apical isosmotic’ condition by the replacement of the apical solution. *P*_*Na*_ was decreased and *P*_*Cl*_ was increased gradually with time under the ‘apical hyposmotic’ condition, and the elimination of the osmotic gradient at time 120 min promptly restored the decreased cation selectivity. N = 4. (B) Immunofluorescence microscopy for claudin-2 and ZO-1. Epithelia were fixed at 120 min after the apical hyposmolality (120 min) and 10 and 60 min after the elimination of the osmotic gradient (130 min and 180 min). The protruded signals of claudin-2 and ZO-1 from the lines of cell-cell contacts were observed at 120 min after the apical hyposmolality (120 min; *arrowheads*), and these protrusions disappeared at 10 min after the elimination of the osmotic gradient (130 min). The jagged shape of cell-cell contacts was slightly recovered at 60 min after the elimination of the osmotic gradient (180 min). Scale bar = 5 μm. (C) Scanning electron microscopy of MDCK II cells. Epithelia were fixed at each time point. Globular structures were observed around cell-cell contacts at 120 min after the apical hyposmolality (*arrowheads*), and these structures disappeared at 10 min after the elimination of the osmotic gradient (130 min). Scale bar = 2 μm.

Next, we investigated the reversibility of the changes induced by the apical hyposmolality. MDCK II cells were incubated under the ‘apical hyposmotic’ condition for 120 min, and then the condition was changed to the ‘apical isosmotic’ condition by the replacement of the apical solution. The *P*_*Na*_ decreased and *P*_*Cl*_ increased gradually with time under the ‘apical hyposmotic’ condition similar to the results in Fig 1. The elimination of the osmotic gradient at time 120 min promptly restored the decreased cation selectivity (*P*_*Na*_/*P*_*Cl*_ at time 5 min, 22.41 ± 0.94; time 120 min, 3.40 ± 0.10; time 125 min, 16.55 ± 0.38), although the value of *P*_*Na*_ was significantly lower than that immediate after the apical hyposmolality (*P*_*Na*_ at time 5 min, 46.20 ± 1.83 × 10^−6^ cm/s; time 120 min, 33.46 ± 1.03 × 10^−6^ cm/s; time 125 min, 27.13 ± 0.56 × 10^−6^ cm/s; Fig 7A). In immunofluorescence microscopy, the protruded signals of claudin-2 and ZO-1 from the lines of cell-cell contacts were observed at time 120 min, and these protrusions disappeared at 10 min after the elimination of the osmotic gradient (time 130 min). The jagged shape of cell-cell contacts was slightly recovered at 60 min after the elimination of osmotic gradient (time 180 min; Fig 7B). In scanning electron microscopy, the blebs induced by the ‘apical hyposmotic’ condition also disappeared after the elimination of osmotic gradient (Fig 7C). These results indicate the reversibility of the decrease of cation selectivity and bleb formation under the apical hyposmolality.

### Effects of apical hyposmolality in claudin-2 expressing MDCK I cells

**Fig 8:**
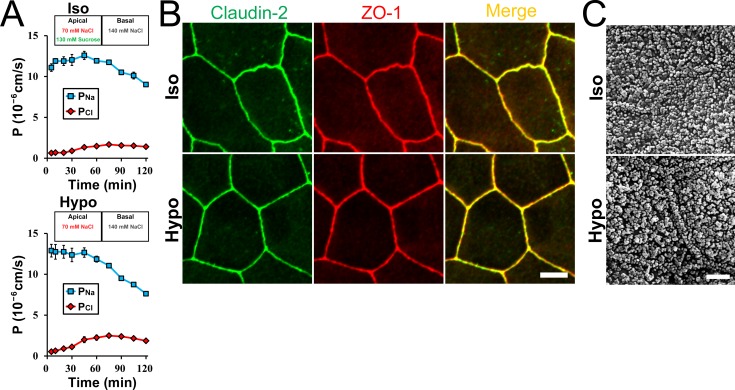
Effects of apical hyposmolality in claudin-2 expressing MDCK I cells. (A) Time course of *P*_*Na*_ and *P*_*Cl*_ in claudin-2 expressing MDCK I cell clone established in a previous study [19]. Under the ‘apical hyposmotic’ condition (Hypo), the decrease of *P*_*Na*_ and the increase of *P*_*Cl*_ were more remarkable than those under the ‘apical isosmotic’ condition (Iso). N = 3 for each experiment. (B) Immunofluorescence microscopy for claudin-2 and ZO-1. The epithelia were fixed 30 min after the replacement of the solutions. Scale bar = 5 μm. (C) Scanning electron microscopy of MDCK I cells expressing claudin-2. Scale bar = 2 μm.

Claudin-2 is a major determinant of high transepithelial conductance in MDCK II cells, and values of *P*_*Na*_ and *P*_*Cl*_ in claudin-2 knockout MDCK II cells have been reported to be approximately 1% and 12% of those in wild-type MDCK II cells, respectively [22]. Therefore, claudin-2 is thought to be responsible for most of *P*_*Na*_ and *P*_*Cl*_, and the decrease of cation selectivity in MDCK II cells by the apical hyposmolality is likely to be dependent on claudin-2. MDCK I cells lack the endogenous expression of claudin-2, and the exogenous expression of claudin-2 in MDCK I cells has been reported to increase transepithelial conductance and cation selectivity [18,19]. Then, we investigated the effects of apical hyposmolality in MDCK I cells. In wild-type MDCK I cells, the value of *P*_*Na*_ was slightly higher than *P*_*Cl*_, and the values were almost constant during 120 min of incubation under the ‘apical hyposmotic’ condition (S6 Fig). In contrast, basal hyposmolality increased *P*_*Na*_ more selectively than *P*_*Cl*_ in wild-type MDCK I cells. Then, we investigated the effects of apical hyposmolality on MDCK I cells expressing exogenous claudin-2. MDCK I cell clones expressing claudin-2 were established in previous studies and used for the experiments in Fig 8 [19] and S7 Fig [22]. Under the ‘apical isosmotic’ condition, the value of *P*_*Na*_ was much higher than *P*_*Cl*_ immediately after the replacement of the apical solution in MDCK I cells expressing claudin-2, and the value of *P*_*Na*_ was decreased and *P*_*Cl*_ was increased slightly with time (Fig 8A and S7A Fig). In contrast, under the ‘apical hyposmotic’ condition, the values of *P*_*Na*_ was also much higher than *P*_*Cl*_ immediately after the replacement of the apical solution, and the decrease of *P*_*Na*_ and the increase of *P*_*Cl*_ during the incubation were more remarkable than those under the ‘apical isosmotic’ condition. The signals of claudin-2 and ZO-1 at apical junctional levels under the ‘apical isosmotic’ condition showed a linear shape of cell-cell contacts in MDCK I cells. The shape of cell-cell contacts and the localization of claudin-2 and ZO-1 under the ‘apical hyposmotic’ condition were similar to those under the ‘apical isosmotic’ condition (Fig 8B and S7B Fig), and bleb formation was not observed under the ‘apical hyposmotic’ condition in scanning electron microscopy (Fig 8C and S7C Fig). These results indicate that apical hyposmolality induces the decrease of cation selectivity with no obvious effects on the structure at cell-cell contacts in MDCK I cells expressing claudin-2.

### Effects of apical hyposmolality in claudin-2 knockout MDCK II cells

**Fig 9:**
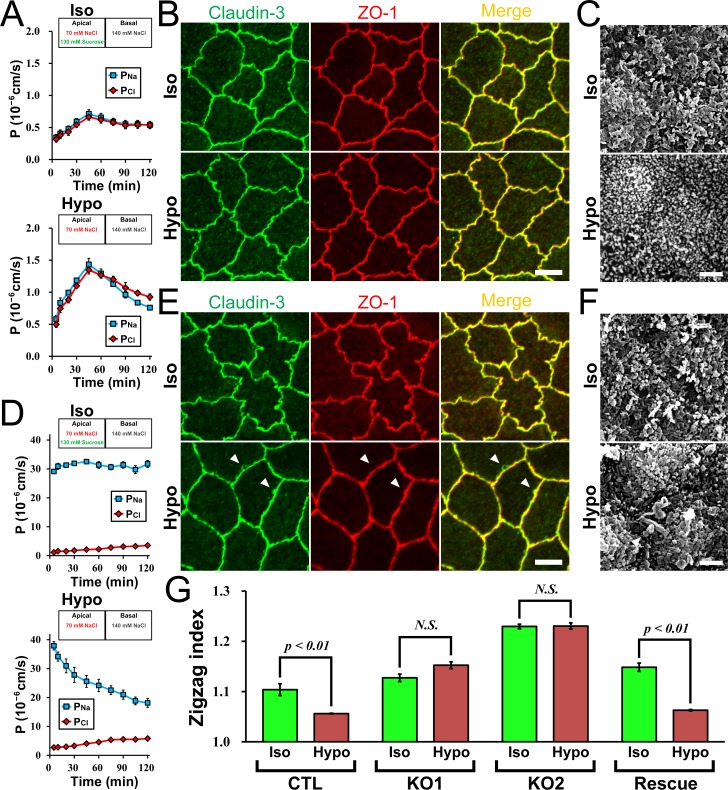
Effects of apical hyposmolality in claudin-2 knockout MDCK II cells. (A) Time course of *P*_*Na*_ and *P*_*Cl*_ in claudin-2 knockout MDCK II cell clone (knockout clone 1 in a previous study [22]). The values of *P*_*Na*_ and *P*_*Cl*_ were increased and then decreased during 120 min of incubation, and the charge selectivity showed no remarkable change under the ‘apical hyposmotic’ condition. N = 4 for each experiment. (B) Immunofluorescence microscopy for claudin-3 and ZO-1. The signals of claudin-3 and ZO-1 under the ‘apical hyposmotic’ condition were similar to those under the ‘apical isosmotic’ condition. Scale bar = 5 μm. (C) Scanning electron microscopy of claudin-2 knockout MDCK II cells. Scale bar = 2 μm. (D) Time course of *P*_*Na*_ and *P*_*Cl*_ in claudin-2 knockout MDCK II cells expressing exogenous claudin-2. The clones were established from the claudin-2 knockout clone 1 as described in a previous study (F4 clone in a previous study [22]). *P*_*Na*_ was decreased and *P*_*Cl*_ was increased gradually with time under the ‘apical hyposmotic’ condition. N = 3 for each experiment. (E) Immunofluorescence microscopy for claudin-3 and ZO-1. The protruded signals of claudin-3 and ZO-1 from the lines of cell-cell contacts were observed under the ‘apical hyposmotic’ condition (*arrowheads*). Scale bar = 5 μm. (F) Scanning electron microscopy of claudin-2 knockout MDCK II cells expressing exogenous claudin-2. Globular structures were observed around cell-cell contacts under the ‘apical hyposmotic’ condition (*arrowheads*). Scale bar = 2 μm. (G) Effects of apical hyposmolality on the shape of cell-cell contacts in wild-type and claudin-2 knockout MDCK II cells. The degree of zigzag (zigzag index) was quantified as described in *Materials and Methods* in wild-type MDCK II cells, claudin-2 knockout clones (knockout clone 1 and 2 in a previous study [22]), and a claudin-2 knockout clone expressing exogenous claudin-2 (F4 clone in a previous study [22]). The zigzag index was significantly decreased under the ‘apical hyposmotic’ condition compared with the ‘apical isosmotic’ condition in wild-type MDCK II cells (CTL) and claudin-2 knockout MDCK II cells expressing exogenous claudin-2 (Rescue), and these decreases of zigzag index were not observed in claudin-2 knockout clones (KO 1 and KO 2). N = 3–4 for each experiment.

To examine the role of claudin-2 in the decrease of cation selectivity by the apical hyposmolality in further detail, we investigated the effects of apical hyposmolality in claudin-2 knockout MDCK II cells. We used claudin-2 knockout MDCK II cell clones established in a previous study (knockout clone 1 for the experiment in Fig 9 and knockout clone 2 for the experiment in S8 Fig) [22]. Under the ‘apical isosmotic’ condition, the values of *P*_*Na*_ and *P*_*Cl*_ immediately after the replacement of the apical solution in claudin-2 knockout cells were much lower than those in wild-type MDCK II cells, and the values were increased slightly over time (Fig 9A and S8A Fig). Under the ‘apical hyposmotic’ condition, the values of *P*_*Na*_ and *P*_*Cl*_ were increased and then decreased during 120 min of incubation, and charge selectivity showed no remarkable change in claudin-2 knockout cells. Immunofluorescence microscopy and scanning electron microscopy showed a similar appearance of cell-cell contacts and cell surface in claudin-2 knockout MDCK II cells compared with those in wild-type MDCK II cells under the ‘apical isosmotic’ condition, and these appearances were not changed by the apical hyposmolality in claudin-2 knockout cells. The cell-cell contacts showed a similar jagged shape, and protruded signals of TJ proteins and bleb formation were not observed under the ‘apical hyposmotic’ condition (Fig 9B and 9C and S8 Fig). We also quantified the degree of zigzag in cell-cell contacts (zigzag index) in a similar manner as described previously [25]. The zigzag index was significantly decreased by the apical hyposmolality in wild-type MDCK II cells, and the decrease of the zigzag index by the apical hyposmolality was not observed in claudin-2 knockout clones (Fig 9G). We also performed rescue experiments using the clone established in a previous study [22]. Claudin-2 re-expression restored the response of the decrease of cation selectivity, the change in a shape of cell-cell contacts, and the bleb formation under the apical hyposmolality (Fig 9D–9F). These results indicate that claudin-2 plays important roles in the change in a shape of cell-cell contacts and bleb formation as well as a decrease of cation selectivity under the apical hyposmolality in MDCK II cells.

## Discussion

In this study, we found that the relative apical hyposmolality decreases cation selectivity in MDCK II cell. In 1983, Madara reported that apical hyperosmolality increases transepithelial electrical resistance (TER) and decreases cation selectivity concomitantly with an increase of TJ strands in guinea pig jejunum [5]. After that, however, there have not been many reports that studied the effects of osmolality on the paracellular transport [26–33]. In addition, most of these reports showed that apical osmotic changes decreased TER but did not measure charge selectivity; therefore, the reduction in the barrier in these reports might result simply from disruption of cell-cell contacts. We previously found that basal relative hyposmolality increases *P*_*Na*_ more selectively than *P*_*Cl*_ in the presence of transcellular channel blockers in toad renal tubule cell line (A6 cells) [34,35], but it was difficult to analyze paracellular transport in ‘tight’ epithelia such as A6 cells. Since MDCK II cells are ‘leaky’ epithelia and the property of paracellular transport is well studied, we chose MDCK II cells for the analysis in this study. We found that osmotic gradient also plays an important role in the acute regulation of paracellular transport in MDCK II cells. It should be noted that the response to the direction of osmotic gradient and the response in the paracellular transport are different among cell types. It is likely that the regulation of the paracellular transport by the osmolality in each epithelial cell type has respective physiological significance dependent on their organs. Further analysis of the effects of osmolality on the paracellular transport in various cultured cells and animals is required for the elucidation of the mechanisms and physiological significance of this phenomenon in future studies.

Apical hyposmolality decreased cation selectivity in the paracellular pathway gradually during 120 min, and the elimination of the osmotic gradient induced surprisingly prompt restoration of the cation selectivity within 5 min. These results indicate that the charge selectivity in the paracellular transport can undergo drastic changes in minutes. The permeability of the paracellular pathway is thought to be determined by the combination of claudin channels expressed in TJ strands [6,9,10]. The dynamic behavior of TJ structure has been shown in live imaging of GFP-tagged claudin-1 expressed in L-fibroblast [36] and fluorescence recovery after photo-bleaching analysis of TJ proteins [37]. However, there are not many examples of the acute regulation of paracellular transport by the physiologic inputs [9,38], and the mechanism of the acute regulation of the claudin channel property is poorly understood. In addition, osmotic changes have been reported to affect claudin expression pattern in euryhaline fishs and cultured cells [11–15], but it seems less likely that the changes in claudin expression pattern caused the rapid change in the cation selectivity by the osmotic changes in this study. Claudin-2 forms high conductive channels with cation selectivity in TJs and is responsible for the most of *P*_*Na*_ and *P*_*Cl*_ in MDCK II cells [16–18,22]. Since the decrease of *P*_*Na*_ and the increase of *P*_*Cl*_ by the apical hyposmolality were also observed in MDCK I cells expressing claudin-2 and these changes were not observed in wild-type MDCK I cells and claudin-2 knockout MDCK II cells, apical hyposmolality is likely to affect the cation selective property of claudin-2 channels, resulting in the rapid regulation of cation selectivity in the paracellular pathway in MDCK II cells. Further analysis using a combination of electrophysiological, morphological, and molecular biological approaches is required to reveal the mechanism of the acute regulation of paracellular transport. Recently, ion flux across individual claudin-2 channels has been detected by the novel patch clamp technique [39]. This technique may be useful for the examination of the claudin-2 channel property under the osmotic gradient in future studies. Claudin-2 is expressed in renal proximal tubules, intestine and liver [40–42]. Therefore, the regulation of claudin-2 property by the osmolality may have physiological role in the regulation of paracellular transport in these organs.

In tight epithelia such as wild-type MDCK I cells and claudin-2 knockout MDCK II cells, it is necessary to be careful when interpreting electrophysiological measurements. There are two routes for the movement of substances across the epithelia: transcellular and paracellular pathways. *P*_*Na*_ and *P*_*Cl*_ are affected by these two pathways, and the contribution of the transcellular pathway to *P*_*Na*_ and *P*_*Cl*_ is relatively larger in tight epithelia than in leaky epithelia [20,21]. Therefore, it is necessary to take into consideration the contribution of transcellular pathway in the measurements of *P*_*Na*_ and *P*_*Cl*_ in wild-type MDCK I cells and claudin-2 knockout MDCK II cells in this study.

Apical hyposmolality induced bleb formation in cell-cell contacts. The osmotic gradient between apical and basal sides is thought to act as a driving force for the water movement between apical and basal sides, and the dynamic behavior of the blebs in time-lapse imaging suggest the possible involvement of the water movement by the osmotic gradient in the bleb formation. Since the blebs were observed in the immediate vicinity of TJ strands, the water movement across TJ strands by the osmotic gradient is likely to be involved in the bleb formation. Claudin-2 is the only claudin that has been demonstrated to have water permeability [43,44], and the mechanism of water transport in claudin channels is still less understood. The bleb formation by the apical hyposmolality was not observed in claudin-2 knockout MDCK II cells, suggesting the involvement of claudin-2 in the bleb formation by the osmotic gradient. Further understanding of the mechanism of water transport in claudin channels may be required for the elucidation of the mechanism of bleb formation by the osmotic gradient.

Interestingly, apical hyposmolality changed the shape of cell-cell contacts from a jagged pattern to a slightly linear pattern in MDCK II cells, and this change was not observed in claudin-2 knockout MDCK II cells. These results suggest that the shape of cell-cell contacts is affected by the osmotic gradient and claudin-2 is likely to be involved in this regulation in MDCK II cells. There are various factors that have been reported to be involved in the regulation of the shape of cell-cell contacts [45–49]; however, to our knowledge, the involvement of claudins in this regulation is not known until now. It is premature to discuss the mechanism in the regulation of the shape of cell-cell contacts by the osmotic gradient. Further analysis of the effects of osmolality on the factors known to be involved in the regulation of the shape of cell-cell contacts is required in future studies.

In conclusion, we found that osmotic gradient plays an important role in the acute regulation of paracellular transport. The understanding of the phenomenon may have important implication in the regulation of sodium homeostasis in the body as well as the physiology of TJs.

## Materials and Methods

### Cells, antibodies, and reagents

The MDCK II cells were provided by Dr. Masayuki Murata [19]. MDCK I cells were provided by the late Dr. Shoichiro Tsukita (Kyoto University) and maintained in our laboratory [19]. Cells were grown in DMEM (high glucose) supplemented with 5% fetal bovine serum. Electrophysiological and morphological experiments were performed on cells cultured for 6 days on 12-mm-diameter Transwell filter inserts with a 0.4-μm pore size (Corning, Corning, NY) unless otherwise noted. The cells were plated at a density of 2 × 10^5^ cells/cm^2^ on filter inserts, and the culture medium was exchanged every day.

Mouse anti-ZO-1 monoclonal antibody (mAb) (T8/754), rat anti-occludin mAb (MOC37), and rabbit anti-claudin-2 polyclonal antibody (pAb) were characterized as described previously [50–52]. Mouse anti-claudin-2 mAb (32–5600), rabbit anti-claudin-3 pAb (34–1700), and Alexa Fluor 488 phalloidin (A12379) were purchased from Invitrogen. The rabbit anti-nonmuscle myosin heavy chain II-B (MHC-B) pAb (PRB-445P) was obtained from Covance. The mouse anti-E-cadherin mAb (ECCD-2; M108) was purchased from Clontech.

### cDNA cloning and plasmid construction

The cDNA encoding dog claudin-2 and mouse occludin [19,53] was cloned into pCAGGS [54] with N-terminal Venus. Synthetic oligonucleotides encoding Lifeact (F-actin marker for higher eukaryotes; ATGGGCGTGGCCGACCTGATCAAGAAGTTCGAGAGCATCAGCAAGGAGGAG) [24] was cloned into pCAGGS with C-terminal Venus. To establish stably expressing clones, the vectors were transfected into cells, and stable clones were selected in standard media supplemented with 500 μg/ml G418.

### Immunocytochemistry

Epithelial cells grown on filter inserts were fixed 30 min after the application of osmotic changes with 10% paraformaldehyde for 2 min at room temperature or in 100% methanol for 10 min at −20°C. Then the filters were permeabilized in a solution of 0.2% (w/v) Triton X-100 (EMD Biosciences) in PBS for 60 min. This was followed by blocking with 2% bovine serum albumin and incubation with a primary Ab and then by a fluorescence-labeled secondary Ab. The filamentous actin (F-actin) was visualized using Alexa Fluor 488 phalloidin. The samples were imaged on a Zeiss LSM700 confocal microscope using a 63× Plan Apo lens. Contrast adjustment was generated using Adobe Photoshop (ver. 7.0).

### Immunoblotting

The Laemmli SDS sample buffer was added to the filter inserts. Then the epithelial cells on filter inserts were scraped in the sample buffer, and the buffer was collected to the microtube and boiled for 5 min. For the analysis of the effects of osmolality, the cells were collected 120 min after the application of osmotic changes. The proteins were then separated using the one-dimensional SDS-PAGE and electrotransferred from the gels to PVDF membranes followed by the incubation with primary Abs. The bound Abs was detected via HRP-linked secondary Abs and visualized by enhanced chemiluminescence (ECL Prime Kit; GE Healthcare).

### Electrophysiological measurements

Electrophysiological studies were performed as described previously [22]. In brief, apical and basal medium were replaced to solution A [140 mM NaCl, 5 mM glucose, 5 mM KCl, 1 mM MgCl_2_, 1 mM CaCl_2_, and 10 mM HEPES-NaOH (pH 7.4)] at 37°C. After the confirmation of stability in TER, apical and/or basal solutions were replaced with the solutions containing 70, 140 or 210 mM NaCl with or without 130 mM sucrose or mannitol. The other composition of these solutions was the same as solution A. Electrical resistance and potentials across the epithelia were measured using Millicell-ERS epithelial volt-ohm meter (Millipore). To reduce the fluctuation of liquid junction potential after the replacement of solutions, the electrodes were immersed in the solutions of the experimental condition more than 30 min before the replacement. To determine the ion permeability of Na^+^ (*P*_Na_) and Cl^−^ (*P*_Cl_) across the epithelia, the potentials and TER of cell monolayers were measured in the presence of NaCl concentration gradient between apical and basal sides, and the electrical resistance and potentials of a blank filter under the same condition were subtracted at each time point. In the experiment to confirm the reversibility of the changes by the apical hyposmolality, NaCl concentration in the solution replaced at 120 min after the apical hyposmolality was not changed to avoid the fluctuation of liquid junction potentials. The electrical resistance and potentials of the blank filter were confirmed to be almost constant before and after the solution replacement at time 120 min. The *P*_Na_/*P*_Cl_ ratio was calculated from dilution potentials using the Goldman–Hodgkin–Katz equation. Then the values of *P*_Na_ and *P*_Cl_ were calculated from the TER and *P*_Na_/*P*_Cl_ using the Kimizuka–Koketsu equation [55].

### Transmission electron microscopy

Transmission, scanning and freeze-fracture electron microscopy were performed as described previously [56]. In brief, the epithelial cells grown on filter inserts were fixed 30 min after the application of osmotic changes with 2% paraformaldehyde, 1% glutaraldehyde and 2% tannic acid/0.1 M HEPES buffer for 2 h at room temperature. Then the epithelia were post-fixed with 1% osmium tetroxide/0.1 M HEPES buffer for 60 min at 4°C. Then the samples were dehydrated using increasing concentrations of ethanol (65%, 75%, 85%, 95%, 99%, and 100%) for 10–15 min each. This was followed by the replacement of the ethanol with propylene oxide and the embedment of the epithelia in Poly/Bed 812 epoxy resin (Polyscience). Then the epithelia were sectioned at 70 nm using a diamond knife, and the ultrathin sections were collected on 200-mesh copper grids followed by staining with 1% hafnium chloride in methanol for 1 min and Sato’s lead citrate for 1 min. The samples were observed by a JEM-1011 transmission electron microscope (JEOL).

### Scanning electron microscopy

The epithelial cells grown on filter inserts were fixed 30 min after the application of osmotic changes with 10% paraformaldehyde and 1% glutaraldehyde/0.1 M HEPES buffer for 2 h. Then the cells were post-fixed with 1% osmium tetroxide/0.1 M HEPES buffer for 60 min, followed by the dehydration with ethanol in a similar manner as described in the previous subsection. Next, the ethanol was replaced with t-butanol, and the epithelia were frozen at −20°C followed by the sublimation of t-butanol. The filters were mounted on aluminum planchets with carbon adhesive, and coated with platinum using an ion coater. The samples were then observed by a JSM-6510LVS scanning electron microscope (JEOL).

### Freeze-fracture electron microscopy

The epithelial cells grown on 23-mm diameter Nunc polycarbonate filters with a 0.4-μm pore size (Life Technologies) were fixed 30 min after the application of osmotic changes with 2% paraformaldehyde and 2.5% glutaraldehyde/0.1 M phosphate buffer (pH 7.3) overnight. Then the epithelia were immersed in 30% glycerol/0.1 M phosphate buffer for 1 h and frozen in liquid nitrogen. The frozen samples were fractured at −110°C and shadowed with platinum unidirectionally at an angle of 45° in Balzer’s Freeze Etching system (BAF060, Bal-Tec). The samples were then immersed in household bleach to dissolve the epithelia, and the replicas were picked up on formvar-filmed grids and observed by the JEM-1011 transmission electron microscope.

### Quantification of the degree of zigzag in cell–cell contacts (zigzag index)

Quantification of the degree of zigzag in cell-cell contacts was performed as described previously [25]. In brief, the stacked confocal images of ZO-1 staining at the level of tight junctions were acquired and saved as TIFF files. These files were then opened in ImageJ 1.43u (available at http://rsb.info.nih.gov/ij; developed by Wayne Rasband, National Institutes of Health, Bethesda, MD). The sides of polygonal shapes of cell–cell contacts were determined from the ZO-1 signal, and all sides contained in an area of 815 μm^2^ were manually traced with freehand lines or straight lines. Five areas were randomly captured for each sample. The zigzag index was defined as the ratio of the sum of freehand lines (L_TJ_) to that of straight lines (L_St_) and was calculated as L_TJ_/L_St_. More than 80 sides were analyzed for each sample, and 3–4 samples were analyzed for each clone.

### Time-lapse imaging

Time-lapse imaging was performed as described previously [25]. In brief, MDCK II cells expressing Venus-claudin-2 or Venus-Lifeact were cultured on the reverse side of Transwell filter inserts for 6 days. The time-lapse images of fluorescent Venus signal were collected immediately after the application of osmotic changes every 30 sec for 30 min at 37°C using Metamorph software (version 7.6, Molecular Devices). The software was equipped with an Olympus IX81, and the images were captured using 60× Plan Apo lens through a cooled charge-coupled device camera (ORCA-ER, Hamamatsu).

### Statistical analysis

Data are represented as means ± standard error of the mean. The statistical analysis was performed using the Student’s *t*-test, and *p* < 0.05 was considered statistically significant.

## Supporting Information

S1 Fig Effects of mannitol on the response by the apical hyposmolality in MDCK II cells. (A) Time course of *P*_*Na*_ and *P*_*Cl*_ in MDCK II cells. NaCl concentration in the apical side was decreased by half, and the osmolality was adjusted with mannitol in place of sucrose. The decrease in cation selectivity was also suppressed by the addition of mannitol. N = 4. (B) Immunofluorescence microscopy for claudin-2 and ZO-1. Scale bar = 5 μm. (C) Scanning electron microscopy of MDCK II cells. Scale bar = 2 μm.
(TIF)

S2 Fig Effects of osmolality on the localization of claudin-2 in MDCK II cells. Immunofluorescence microscopy for claudin-2 at low magnification under the osmotic changes. Scale bar = 10 μm.
(TIF)

S3 Fig Effects of osmolality on the surface structure of MDCK II cells. Scanning electron microscopy of MDCK II cells at low magnification under the osmotic changes. Scale bar = 5 μm.
(TIF)

S4 Fig Scanning electron microscopy of MDCK II cells under the osmotic changes. Epithelia were fixed 30 min after the osmotic changes and observed by scanning electron microscopy. Globular structures were observed around cell-cell contacts under the basal hyperosmolality (*arrowheads*). Scale bar = 2 μm.
(TIF)

S5 Fig Time-lapse imaging of occludin under the apical hyposmolality. The images of fluorescent Venus signal were collected before and 120 min after the apical hyposmolality. Scale bar = 5 μm.
(TIF)

S6 Fig Effects of hyposmolality on the barrier function in MDCK I cells. Time course of *P*_*Na*_ and *P*_*Cl*_ in MDCK I cells. Basal hyposmolality increased *P*_*Na*_ more selectively than *P*_*Cl*_. N = 2–3 for each experiment.
(TIF)

S7 Fig Effects of apical hyposmolality in claudin-2 expressing MDCK I cells. (A) Time course of *P*_*Na*_ and *P*_*Cl*_ in claudin-2 expressing MDCK I cell clone established in a previous study [22]. N = 3 for each experiment. (B) Immunofluorescence microscopy for claudin-2 and ZO-1. Scale bar = 5 μm. (C) Scanning electron microscopy of MDCK I cells expressing claudin-2. Scale bar = 2 μm.
(TIF)

S8 Fig Effects of apical hyposmolality in claudin-2 knockout MDCK II cells. (A) Time course of *P*_*Na*_ and *P*_*Cl*_ in claudin-2 knockout MDCK II cell clone (knockout clone 2 in a previous study [22]). N = 3 for each experiment. (B) Immunofluorescence microscopy for claudin-3 and ZO-1. Scale bar = 5 μm. (C) Scanning electron microscopy of claudin-2 knockout MDCK II cells. Scale bar = 2 μm.
(TIF)

S1 Movie Time-lapse imaging of Venus claudin-2 in MDCK II cells under the ‘apical isosmotic’ condition. The images of fluorescent Venus signal were collected immediately after the application of osmotic changes every 30 sec. The Venus signal of claudin-2 showed modest sequential changes during 30 min of the observation.
(AVI)

S2 Movie Time-lapse imaging of Venus claudin-2 in MDCK II cells under the ‘apical hyposmotic’ condition. The signal of claudin-2 showed the occurrence of low signal circular structures at various regions in cell-cell contacts, and these structures expanded to a diameter of about one to three μm and then disappeared within 30 sec to several minutes.
(AVI)

S3 Movie Time-lapse imaging of Venus claudin-2 in MDCK II cells under the ‘apical hyposmotic’ condition. The signal of claudin-2 showed dynamic changes similar to those observed in S2 Movie.
(AVI)

S4 Movie Time-lapse imaging of Venus Lifeact in MDCK II cells under the ‘apical isosmotic’ condition. The Venus signal of Lifeact showed modest sequential changes during 30 min of the observation.
(AVI)

S5 Movie Time-lapse imaging of Venus Lifeact in MDCK II cells under the ‘apical hyposmotic’ condition. The signal of Lifeact showed dynamic changes similar to those observed in claudin-2, although the signal intensity in the circular structures was high.
(AVI)

S6 Movie Time-lapse imaging of Venus Lifeact in MDCK II cells under the ‘apical hyposmotic’ condition. The signal of Lifeact showed dynamic changes similar to those observed in S5 Movie.
(AVI)
