# Supplementary figures and images for: Effects of Osmolality on Paracellular Transport in MDCK II Cells
Source: PLoS One. 2016 Nov 17;11(11):e0166904. doi: 10.1371/journal.pone.0166904 (PMC5113991; doi:10.1371/journal.pone.0166904)

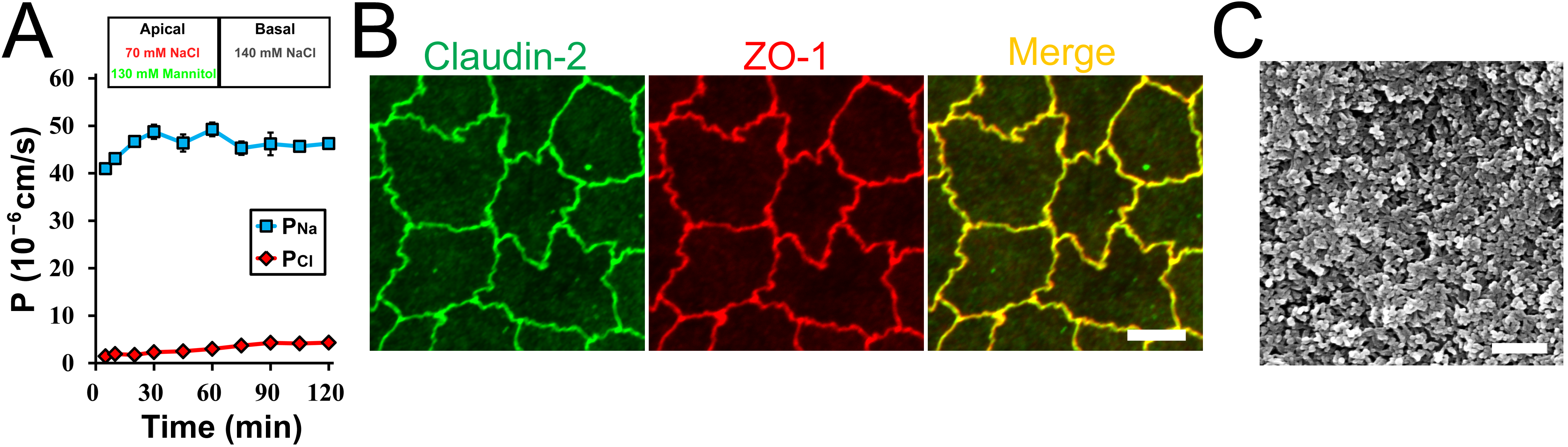

Supplement: S1 Fig — (A) Time course of PNa and PCl in MDCK II cells. NaCl concentration in the apical side was decreased by half, and the osmolality was adjusted with mannitol in place of sucrose. The decrease in cation selectivity was also suppressed by the addition of mannitol. N = 4. (B) Immunofluorescence microscopy for claudin-2 and ZO-1. Scale bar = 5 μm. (C) Scanning electron microscopy of MDCK II cells. Scale bar = 2 μm. (TIF) [file pone.0166904.s001.TIF]

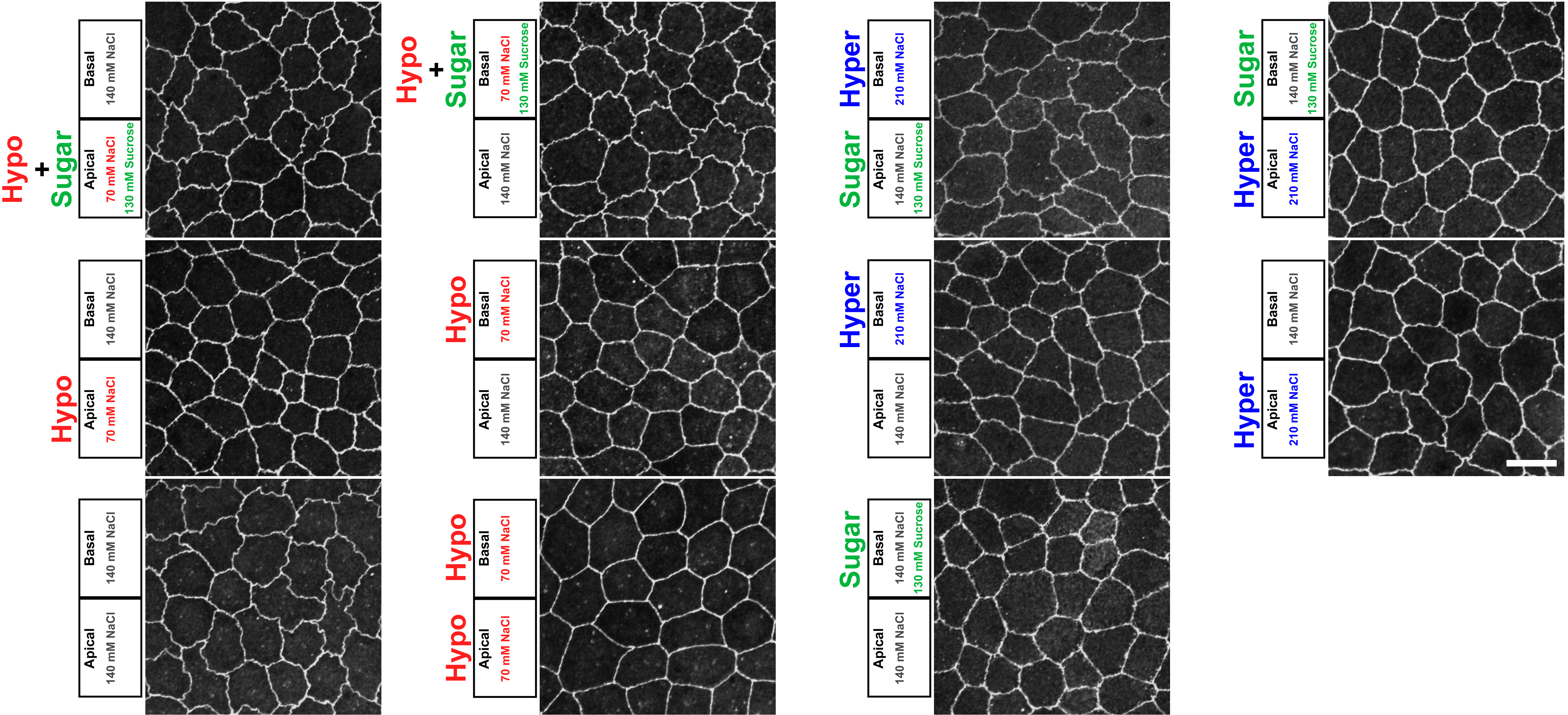

Supplement: S2 Fig — Immunofluorescence microscopy for claudin-2 at low magnification under the osmotic changes. Scale bar = 10 μm. (TIF) [file pone.0166904.s002.TIF]

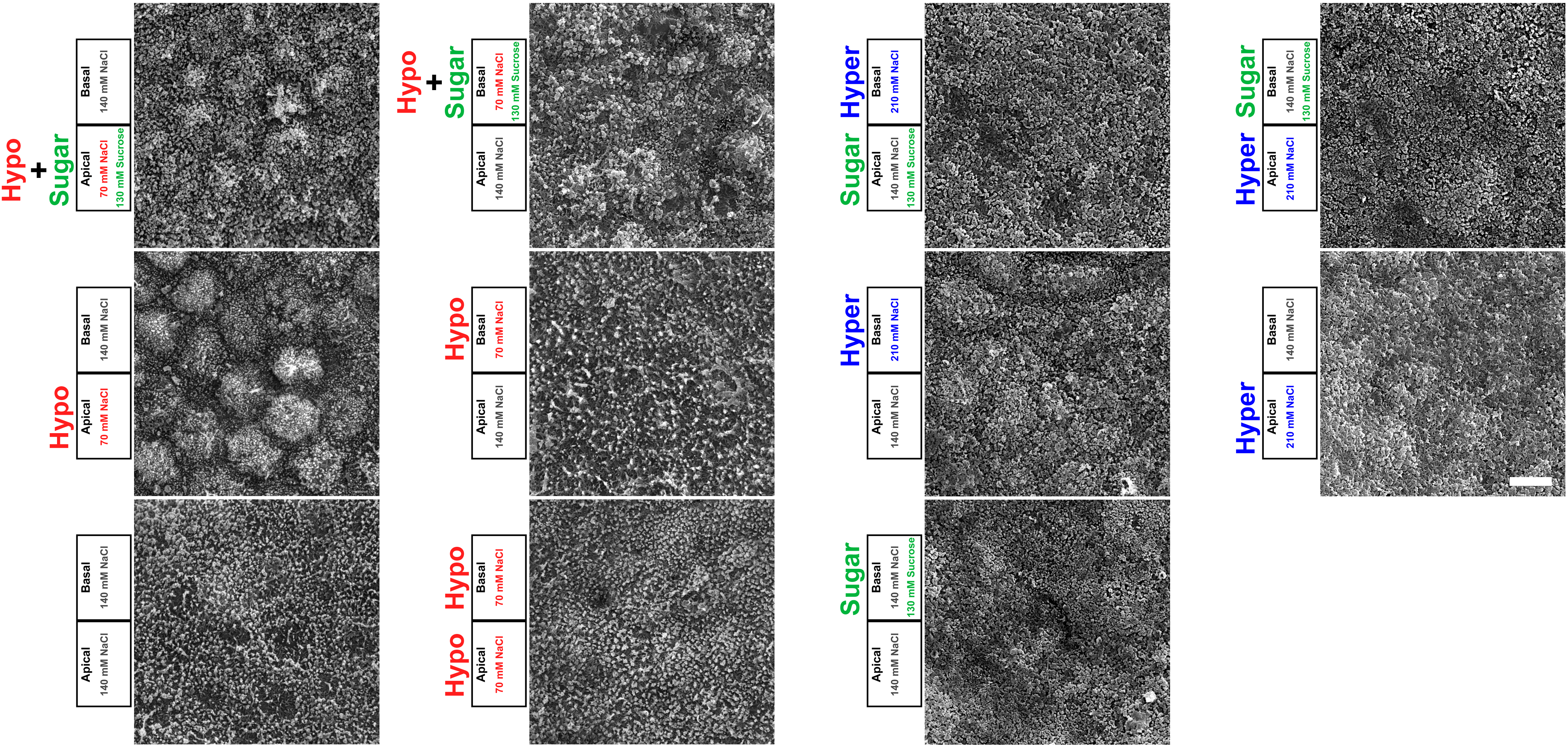

Supplement: S3 Fig — Scanning electron microscopy of MDCK II cells at low magnification under the osmotic changes. Scale bar = 5 μm. (TIF) [file pone.0166904.s003.TIF]

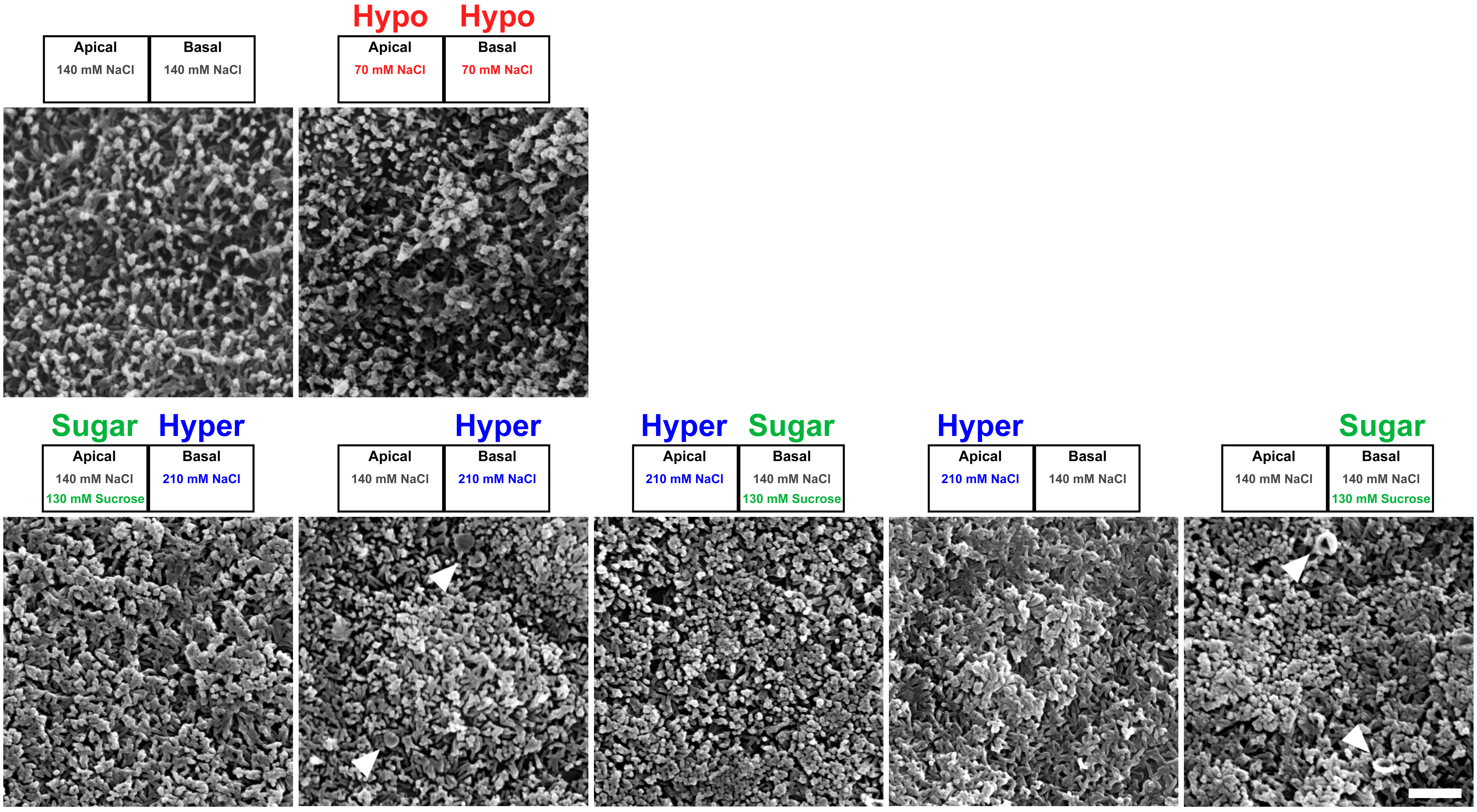

Supplement: S4 Fig — Epithelia were fixed 30 min after the osmotic changes and observed by scanning electron microscopy. Globular structures were observed around cell-cell contacts under the basal hyperosmolality (arrowheads). Scale bar = 2 μm. (TIF) [file pone.0166904.s004.TIF]

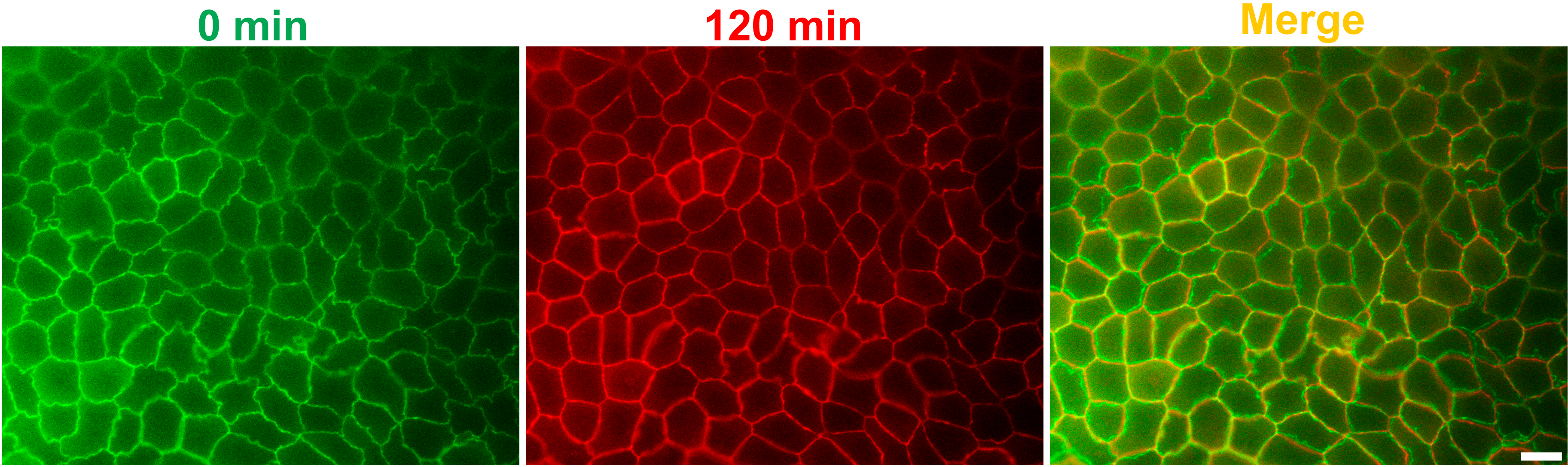

Supplement: S5 Fig — The images of fluorescent Venus signal were collected before and 120 min after the apical hyposmolality. Scale bar = 5 μm. (TIF) [file pone.0166904.s005.TIF]

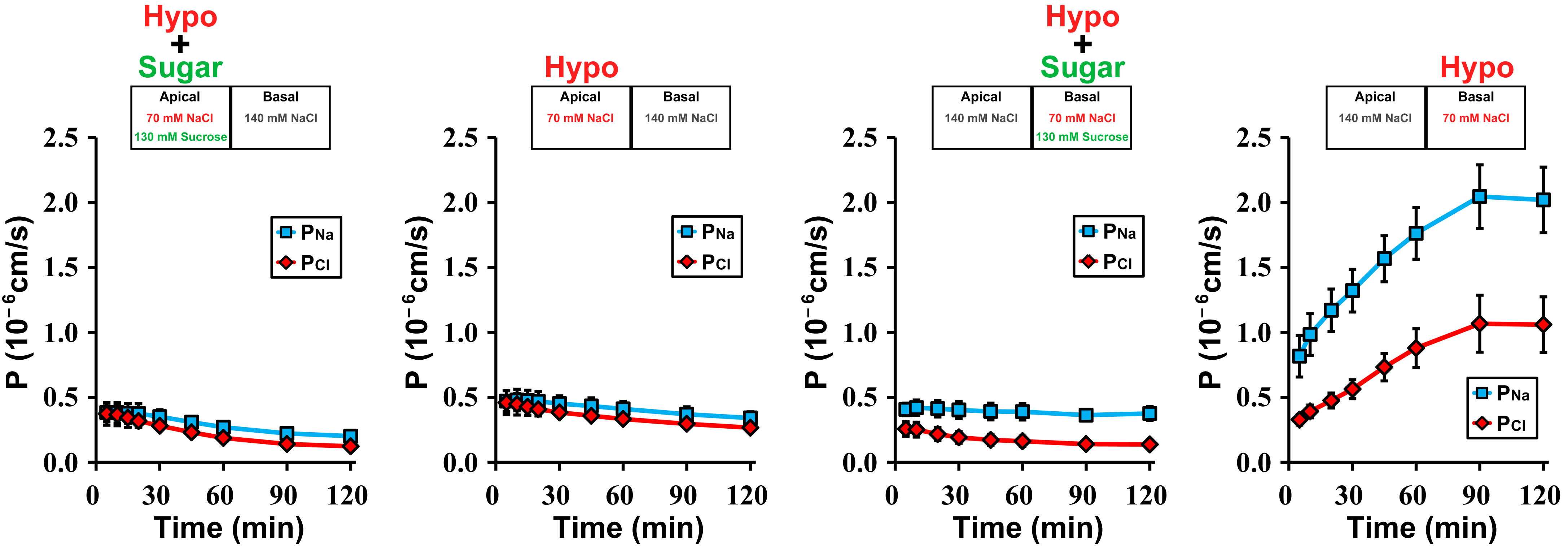

Supplement: S6 Fig — Time course of PNa and PCl in MDCK I cells. Basal hyposmolality increased PNa more selectively than PCl. N = 2–3 for each experiment. (TIF) [file pone.0166904.s006.TIF]

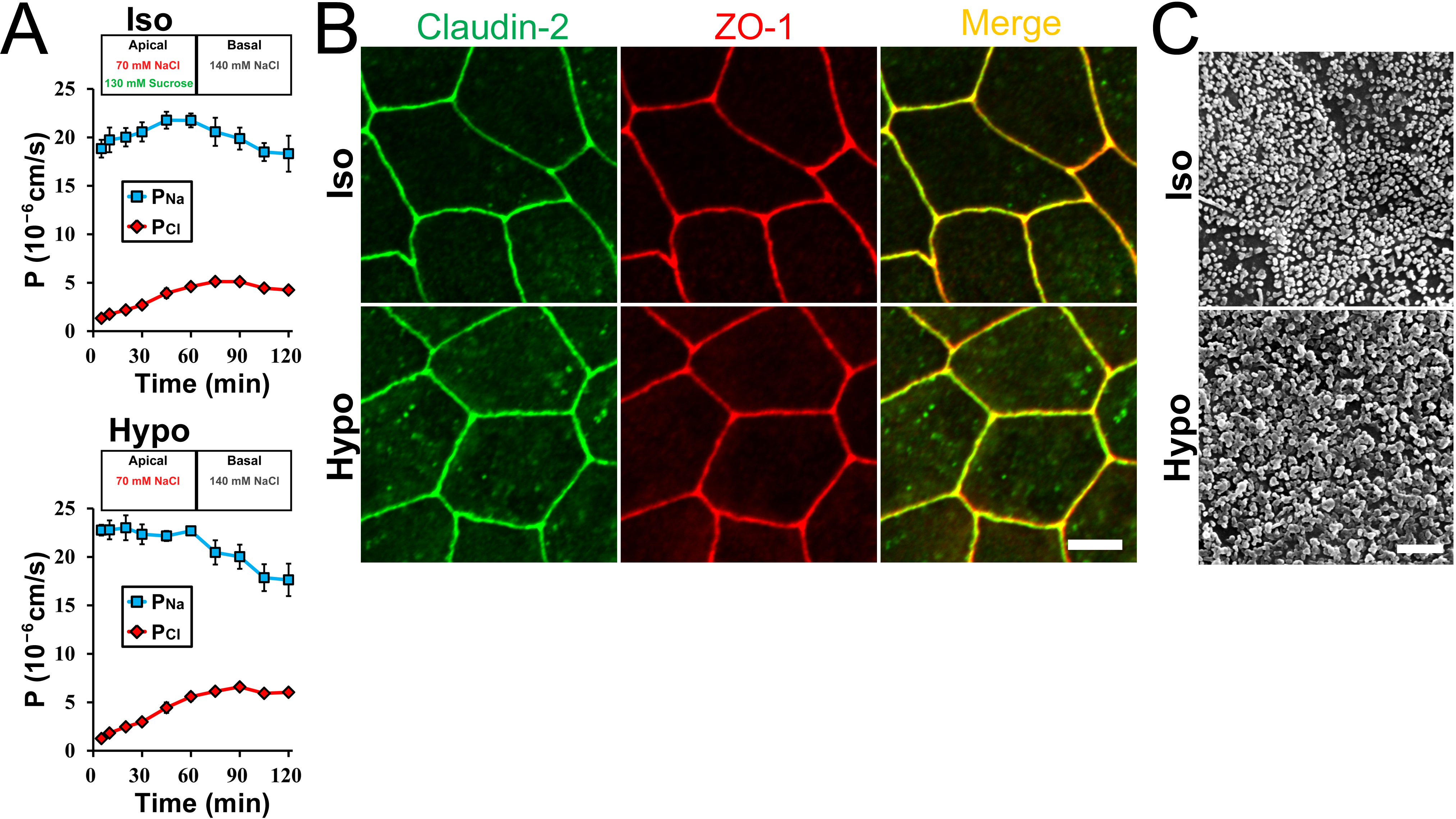

Supplement: S7 Fig — (A) Time course of PNa and PCl in claudin-2 expressing MDCK I cell clone established in a previous study [22]. N = 3 for each experiment. (B) Immunofluorescence microscopy for claudin-2 and ZO-1. Scale bar = 5 μm. (C) Scanning electron microscopy of MDCK I cells expressing claudin-2. Scale bar = 2 μm. (TIF) [file pone.0166904.s007.TIF]

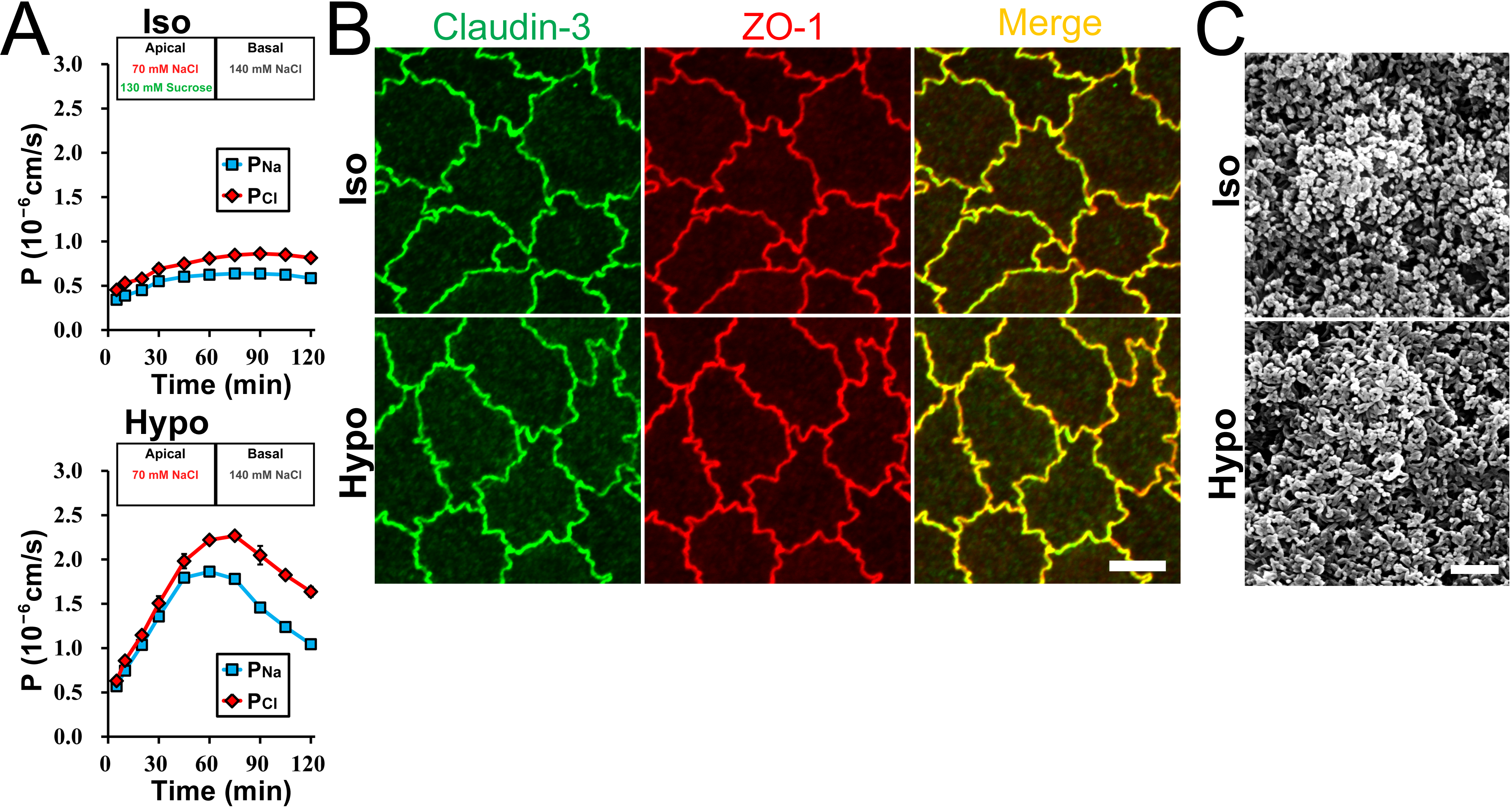

Supplement: S8 Fig — (A) Time course of PNa and PCl in claudin-2 knockout MDCK II cell clone (knockout clone 2 in a previous study [22]). N = 3 for each experiment. (B) Immunofluorescence microscopy for claudin-3 and ZO-1. Scale bar = 5 μm. (C) Scanning electron microscopy of claudin-2 knockout MDCK II cells. Scale bar = 2 μm. (TIF) [file pone.0166904.s008.TIF]
